# Dystrophin- and Utrophin-Based Therapeutic Approaches for Treatment of Duchenne Muscular Dystrophy: A Comparative Review

**DOI:** 10.1007/s40259-023-00632-3

**Published:** 2023-11-02

**Authors:** Sylwia Szwec, Zuzanna Kapłucha, Jeffrey S. Chamberlain, Patryk Konieczny

**Affiliations:** 1 Institute of Human Biology and Evolution, Faculty of Biology, Adam Mickiewicz University, ul. Uniwersytetu Poznańskiego 6, 61-614 Poznań, Poland; 2 Department of Neurology, University of Washington School of Medicine, Seattle, WA 98109-8055 USA; 3 Senator Paul D. Wellstone Muscular Dystrophy Specialized Research Center, University of Washington School of Medicine, Seattle, WA 98109-8055 USA; 4 Department of Biochemistry, University of Washington School of Medicine, Seattle, WA 98109-8055 USA; 5 Department of Medicine, University of Washington School of Medicine, Seattle, WA 98109-8055 USA

## Abstract

Duchenne muscular dystrophy is a devastating disease that leads to progressive muscle loss and premature death. While medical management focuses mostly on symptomatic treatment, decades of research have resulted in first therapeutics able to restore the affected reading frame of dystrophin transcripts or induce synthesis of a truncated dystrophin protein from a vector, with other strategies based on gene therapy and cell signaling in preclinical or clinical development. Nevertheless, recent reports show that potentially therapeutic dystrophins can be immunogenic in patients. This raises the question of whether a dystrophin paralog, utrophin, could be a more suitable therapeutic protein. Here, we compare dystrophin and utrophin amino acid sequences and structures, combining published data with our extended in silico analyses. We then discuss these results in the context of therapeutic approaches for Duchenne muscular dystrophy. Specifically, we focus on strategies based on delivery of micro-dystrophin and micro-utrophin genes with recombinant adeno-associated viral vectors, exon skipping of the mutated dystrophin pre-mRNAs, reading through termination codons with small molecules that mask premature stop codons, dystrophin gene repair by clustered regularly interspaced short palindromic repeats (CRISPR)/CRISPR-associated protein 9 (CRISPR/Cas9)-mediated genetic engineering, and increasing utrophin levels. Our analyses highlight the importance of various dystrophin and utrophin domains in Duchenne muscular dystrophy treatment, providing insights into designing novel therapeutic compounds with improved efficacy and decreased immunoreactivity. While the necessary actin and β-dystroglycan binding sites are present in both proteins, important functional distinctions can be identified in these domains and some other parts of truncated dystrophins might need redesigning due to their potentially immunogenic qualities. Alternatively, therapies based on utrophins might provide a safer and more effective approach.

## Key Points

**Table:** 

Drugs based on restoration of the reading frame of dystrophin via readthrough of nonsense codons and antisense oligonucleotide-driven exon skipping, as well as recombinant adeno-associated viral vector-mediated delivery of micro-dystrophin are now available in some countries to boys affected with Duchenne muscular dystrophy (DMD).
Beyond immune response against the viral vector, the potentially therapeutic exogenous dystrophins can also be immunogenic in DMD patients, highlighting a limitation of dystrophin gene therapy. Utrophin, an autosomal functional paralog of dystrophin naturally expressed in dystrophic muscles, may therefore represent a viable option, and utrophin-based gene approach offers a potential safer therapeutic solution.
Comparison of amino acid sequences and protein structures of dystrophin and utrophin highlights the importance of distinct regions and domains in the therapeutic outcome.
The H1 and H3 regions that have been commonly used in potentially therapeutic micro-dystrophins have no sequence homology with the corresponding regions of utrophin and therefore could theoretically more easily induce an immune response in boys with DMD.
Either micro-dystrophin or micro-utrophin sequences could be optimized to enhance their therapeutic potential in DMD patients.

## Introduction

**Fig. 1:**
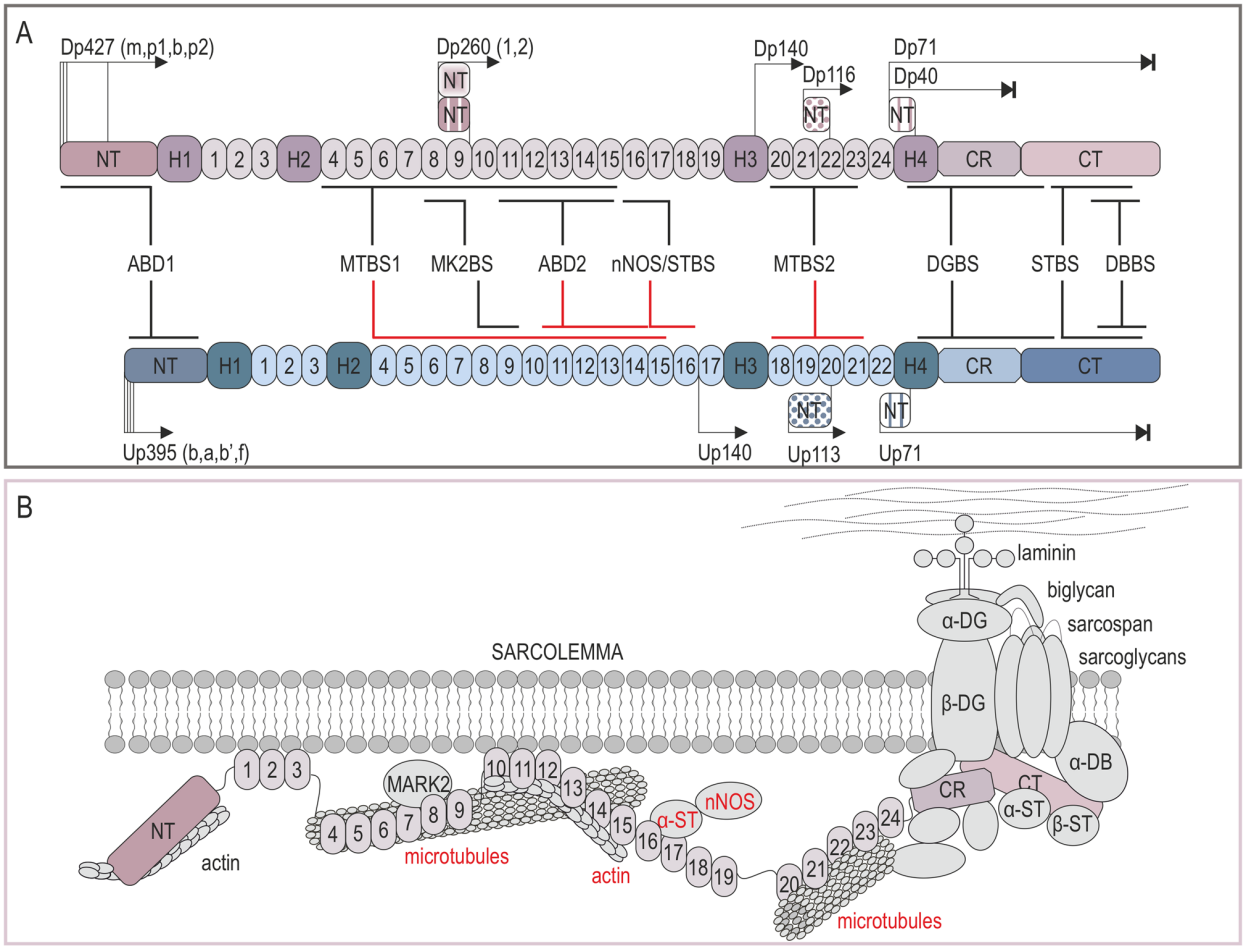
Comparison of binding properties of dystrophin and utrophin. **A** Both the full length Dp427 dystrophin and Up395 utrophin have four main regions: the N-terminal domain (NT), the central rod domain composed of four hinges (H1–H4) and 24/22 spectrin repeats, the cysteine-rich domain (CR), and the C-terminal domain (CT). Red lines note that the binding properties of dystrophin are not retained in utrophin. **B** Dystrophin assembles the dystrophin glycoprotein complex that includes dystroglycans, sarcoglycans, sarcospan, syntrophins, and dystrobrevins, and associate with other proteins as indicated in the figure. Utrophin also assembles the complex but loses the ability to interact with microtubules, actin through ABD2, and nNOS (marked red). *ABD1/2* actin biding domain 1/2, *DBBS* α-dystrobrevin binding site, *DGBS* β-dystroglycan binding site, *MK2BS* Ser/Thr kinase MAP/Microtubule affinity-regulating kinase 2 (MARK2) binding site, *nNOS/STBS* nNOS/α-syntrophin binding site, *MTBS1/2* microtubules binding site 1/2, *STBS* α/β-syntrophins binding site

The dystrophin gene [*DMD*, Online Mendelian Inheritance in Man (OMIM) #300377], covering 2,241,933 bp and approximately 1.5% of the X chromosome, is the longest gene in the human genome [1]. Several mature transcripts are generated from seven biologically significant *DMD* promoters in a tissue and time-dependent manner. These transcripts encode isoforms ranging in molecular mass from 40 kDa to the full-length 427 kDa dystrophin (Dp40–Dp427; Fig. 1A) [2]. Depending on the mutation site, genomic alterations within the *DMD* gene may affect translation of only the longest isoform or also other dystrophins. This correlates with the severity of Duchenne muscular dystrophy (DMD, OMIM #310200) and pathology in tissues, in which specific dystrophins are normally present. The disease affects mainly boys (1/5000) and is manifested by progressive muscle failure and neuropsychiatric symptoms [3]. The first disease indications usually become apparent between the ages of 2–5 years as motor developmental delay and abnormal gait, weakened proximal muscles, and calf muscle pseudohypertrophy are observed. Progressive muscle degradation leads to loss of ambulation at the age of 8–12 years and premature death at around 30 years due to respiratory and cardiac complications [4]. Genomic alterations that do not change the reading frame of the *DMD* gene often result in shorter but partially functional proteins. Correspondingly, these patients show a more benign form of the disease, Becker muscular dystrophy (BMD, OMIM #300376).

Most dystrophins assemble the dystrophin–glycoprotein complex (DGC; Fig. 1B) on the plasmalemma of various cell types, including muscle fibers, satellite cells, cardiomyocytes, and neurons, playing mechanical as well as signaling functions [1, 5–7]. These functions include transmitting forces during contraction by connecting the internal cytoskeleton with the extracellular matrix (muscle cells), establishing cell polarity (satellite cells), maturation of neurotransmitter receptor complexes and their release regulation at the neuro-muscular junctions (NMJs) and central synapses (nervous system), and binding several regulatory proteins important in signal transduction inside the cell and between different cell types. Some dystrophins were also found in non-subplasmalemmal localizations, including the nucleus, mitochondria, and cytoplasm [8, 9].

Currently, management of DMD is based on symptomatic treatment that entails physiotherapy and the use of corticosteroids, including the Food and Drug Administration (FDA)-approved pro-drug Emflaza (deflazacort) [10]. Importantly, decades of research have resulted in first therapeutics aimed to restore the affected reading frame of *DMD* transcripts or induce synthesis of a micro-dystrophin (μDys) protein from a recombinant adeno-associated viral (rAAV) vector, with others, based on gene therapy and cell signaling, in preclinical or clinical development [11]. The latter include delivery of rAAV vectors carrying truncated coding sequences  of a dystrophin paralog, utrophin (μUtr). μDys and μUtr proteins can assemble the DGC and the utrophin–glycoprotein complex (UGC), respectively, and restore the connection between the actin-based cytoskeleton and the extracellular matrix [12, 13]. Other therapeutics in development include pharmaceuticals stimulating expression of the utrophin gene (*UTRN*) or activating muscle regeneration via induction of specific signaling pathways and/or epigenomic modifications [6, 14]. Bearing in mind that most of the prospective approaches for DMD rely on the use of only partially functional truncated dystrophins or utrophins, we compare here the sequence and structure of these proteins, highlighting importance of distinct regions and domains in the therapeutic context.

## Dystrophin Functions

DMD has been classically related to loss of the full-length dystrophin in the striated muscle tissue, where it provides strength, flexibility, and stability to myofibers and cardiomyocytes by influencing focal adhesion tension [15] as well as by acting as a molecular shock absorber and providing protection to the plasmalemma from contraction-induced damage [16, 17]. Other Dp427-related roles include signal transmission in and outside the differentiated muscle cells [6, 14], as well as control of the division dynamics of activated skeletal muscle stem (satellite) cells [5]. Functions of other isoforms are less known, although, e.g., the Dp71 isoform has been indicated to regulate cell proliferation [18].

Generally, dystrophin roles are considered in terms of complexes that they assemble. Most dystrophins can bind β-dystroglycan and assemble the DGC (Fig. 1); however, this interaction and the complex stability may differ dependent on the posttranslational modification and the dystrophin isoform. Specifically, phosphorylation of tyrosine in the dystrophin 15 most C-terminal amino acids was shown to disrupt the dystrophin interaction with β-dystroglycan in C2/C4 myoblasts mouse cell line [19]. In contrast, 3D modeling predicted enhanced dystrophin interaction with β-dystroglycan upon phosphorylation of S3059 at the WW domain [20]. It is plausible that dystrophin phosphorylation regulates the dystrophin interaction with β-dystroglycan, the β-dystroglycan phosphorylation state and stability of the DGC. Indeed, Miller et al. showed that preventing phosphorylation of β-dystroglycan led to restoration of the DGC components at the sarcolemma and amelioration of the dystrophic phenotype in a mouse model of DMD, the *mdx* mouse [21].

Shorter dystrophin isoforms lose the ability to connect specific proteins and, in consequence, the assembled complexes might have different overall roles dependent on the dystrophin isoform. The most extreme example is the shortest dystrophin isoform, Dp40, devoid of the full β-dystroglycan binding site at the N-terminus and the C-terminal (CT) domain. Specifically, Dp40 was shown to localize in neurons to synaptic vesicles rather than to the membrane fraction and interact with a group of presynaptic proteins, including syntaxin1A and SNAP25 [22]. Interestingly, not all β-dystroglycan in skeletal muscle is bound to dystrophin, with the estimated β-dystroglycan to dystrophin molar ratio 40:1 [23]. Other binding partners of β-dystroglycan include cavin-1, calcium channels, or plectin [23, 24]. Additionally, alternative splicing of Dp140, Dp116, and Dp71 might influence binding of syntrophins and dystrobrevins to the complex [25–28].

## Dystrophin Domains

**Fig. 2:**
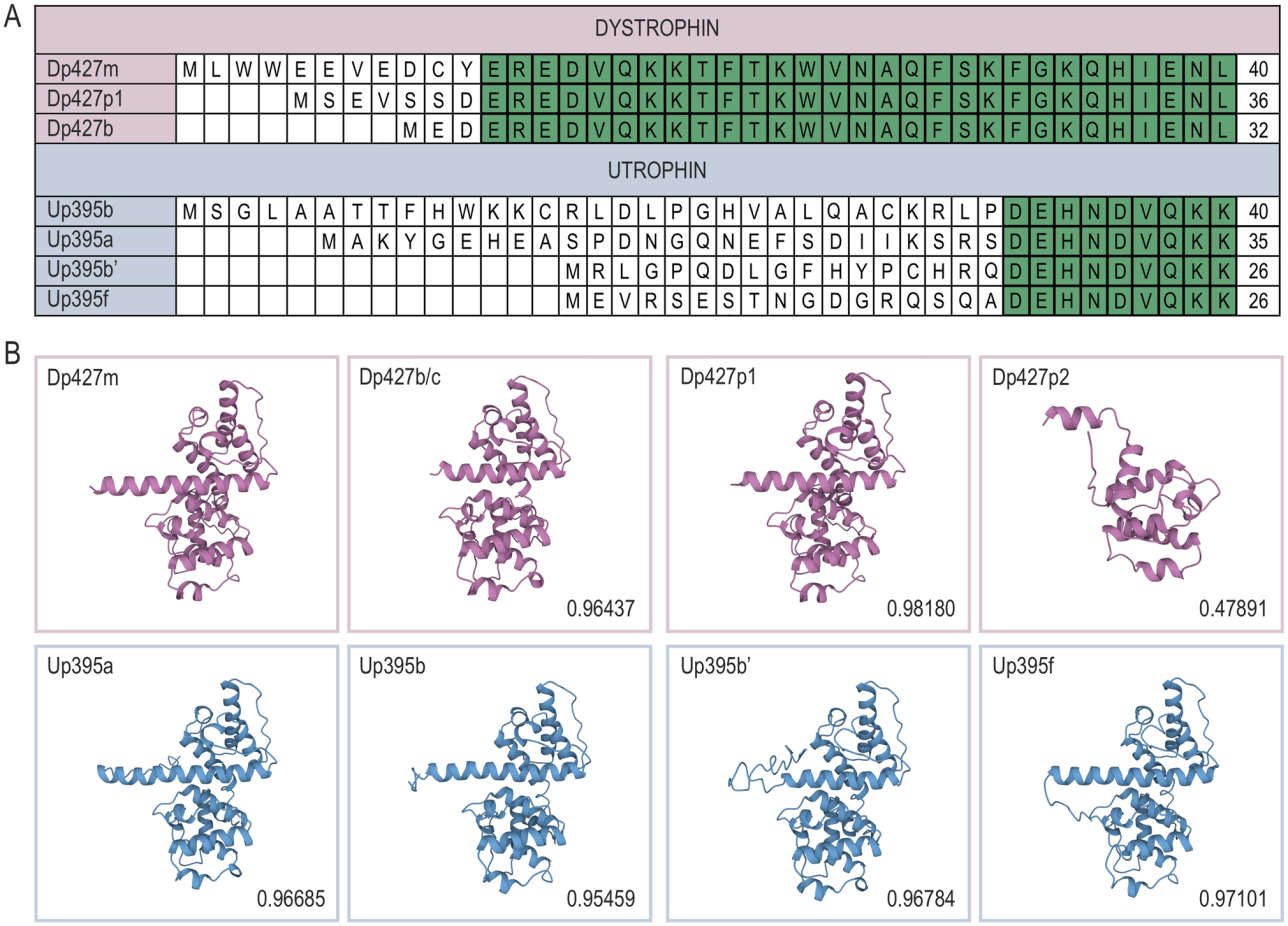
Comparison of ABD1 of the full-length human dystrophin and utrophin variants. Amino acid alignments (**A**) and 3D structures of ABD1 (I-TASSER software) (**B**) were based on the following fragments: Dp427m, 1–246 (NP_003997.2); Dp427p1, 1–242 (NP_004000.1); Dp427p2, 1–123 (NP_004001.1); Dp427b (Dp427c), 1–238 (NP_000100.3), Up395a, 1–261 (NP_009055.2); Up395b, 1–266 (XP_005267184.1); Up395b’, 1–252 (XP_024302304), and Up395f, 1–252 (XP_005267190.1). The structures were compared with Dp427m with the TM-align software. Note, very high TM-scores (above 0.95), except Dp427p2 (0.47891). Note also that transcription of Dp427p1 and p2 begins from the same promoter but the coding sequence of p2 starts from the 124th amino acid (methionine) of Dp427m. The Up395b’ amino acid sequence is marked as putative [64]. Up395a and Up395a’ transcription starts from different promoters but have the same coding sequence

Transcription of Dp427 starts from promoters active mainly in the brain (B), muscle (M), and Purkinje (P) cells [2]. All these mRNAs are estimated to take 10 hours to be generated [29] and give rise to almost identical Dp427 proteins, which differ only in the first several amino acids [2] (Fig. 2A). Dp427 dystrophin consists of four major domains: an N-terminal (NT) domain that constitutes the first actin-binding domain (ABD1; Dp427b, 1–238 aa; Dp427m, 1–246 aa; Dp427p1, 1–242 aa; Dp427p2, 1–123 aa), a central rod (CenR) domain (Dp427m, 253–3112 aa), a cysteine-rich (CR) domain containing 14 cysteines (Dp427m, 3113–3360 aa), and a C-terminal (CT) domain (Dp427m, 3361–3685 aa) [30–32] (Fig. 1). ABD1 contains two calponin homology domains that incorporate three actin binding sites (Dp427m: first, 18–27 aa; second, 88–116 aa; third, 131–147 aa) [32], responsible for binding to F- and γ-actin and linking the sarcolemma to the subsarcolemmal network [1, 33, 34]. Additionally, ABD1 can interact with intermediate filament protein cytokeratins 8/19 that allow dystrophin to associate with the contractile apparatus [35]. In contrast to the globular shape of the ABD1, the CenR domain has a ruler-like α-helical structure, build from 24 tandem spectrin-like repeats (R1–R24) and four proline-rich regions (hinges, H1–H4) positioned before R1, between R3–R4 and R19–R20, and after R24 [31]. R11–R15 contain an anchored second actin-binding motif (ABD2) enriched in basic amino acids, indicative of the electrostatic interaction that underlies the ABD2 binding to the acidic actin filaments [36, 37]. The cooperation of ABD1 and ABD2 is responsible for the strong association of dystrophin with actin filaments [38].

R8–R9 of the CenR domain associate in activated satellite cells with the Ser/Thr kinase MAP/microtubule affinity-regulating kinase 2 (MARK2) also known as partitioning-defective 1b (Par1b) [39], an important regulator of cell polarity, asymmetric divisions, and regenerative processes in skeletal muscle [5, 40]. R16–R17 contain the binding site for neuronal nitric oxide synthase (nNOS) [41, 42], a messenger molecule with multiple roles in modulating various cell functions, including gene transcription and mRNA translation, generation of posttranscriptional modifications, and oxidative metabolism, as well as neurotransmission and vascular tone [43], important in skeletal muscle contraction and function [41, 44, 45]. Notably, recruitment of nNOS to the sarcolemma requires not only dystrophin but also α-syntrophin that either positions nNOS on dystrophin [46] or anchors nNOS to dystrophin through direct binding to R17 [47]. In fact, syntrophins were recently shown to have three putative binding sites in the dystrophin CenR domain and, apart from α-syntrophin interaction with R17, binding of R22 to β1/β2-syntrophins was confirmed by pull-down assays [47]. The CenR domain is also involved in interaction of dystrophin with microtubules through R4–R15 (microtubule binding site 1, MTBS1) and R20–R23 (microtubule binding site 2, MTBS2), which are necessary for the proper organization of the microtubule network in skeletal muscle cells [48, 49] (Fig. 1). Besides these functions, CenR domain also provides a flexible connection between the N- and C-terminus of dystrophin [49].

The fourth hinge (H4; Dp427m, 3041–3112 aa) of the CenR domain contains the WW domain (Dp427m, 3055–3092 aa) involved in protein–protein interactions and along with the adjacent EF-hands (Dp427m, EF1, 3130–3157 aa and EF2, 3178–3206 aa) motif in the CR domain, they form a primary binding site for the carboxyl terminus of β-dystroglycan (β-dystroglycan binding site, DGBS; Dp427, 3054–3271 aa) [50]. In addition to the EF-hands, the ZZ-type zinc finger (ZZ) site (Dp427m, 3307–3354 aa) is also present in the CR domain and the EF–ZZ motif was shown to interact with a cytoskeletal linker protein, plectin [24]. Furthermore, amino acids in the ZZ domain are also required for optimal interaction of dystrophin and β-dystroglycan, with the full DGBS mapped to Dp427 amino acids 3054–3446. More recent results obtained by Hnia et al. indicate that amino acids 3326–3332 within the ZZ domain are a crucial part of the contact region between dystrophin and β-dystroglycan [51]. Interestingly, the ZZ domain was also shown to be important for the nuclear transport of Dp71 containing exon 78 in C2C12 cells [52]. β-Dystroglycan together with dystrophin have a high affinity to ankyrin proteins (ankB and ankG), which are required for sarcolemmal integrity during muscle contraction [53]. AnkB binds to the CR domain of dystrophin while ankG interacts with both the cytoplasmic area of β-dystroglycan and dystrophin, restricting their localization to costameres (Fig. 1B). Importantly, the research showed loss of sarcolemmal dystrophin and β-dystroglycan in adult ankB-depleted muscle, suggesting that ankB as well as ankG are essential for localization and functionality of the DGC in skeletal muscle [53, 54]. The CR domain was also shown to interact with intermediate filament protein synemin [55] and calmodulin in a calcium-dependent manner [56]. Together, these results underscore a crucial role of the CR domain in protein–protein interaction and stabilization of the DGC.

Dystrophin is terminated by the CT domain, containing two α-helices, resembling spectrin repeats present in the central domain. Its structure provides binding sites for α-dystrobrevins (dystrobrevin binding site, DBBS) and α/β-syntrophins (syntrophin binding site, STBS) (Fig. 1, Table 1), delimiting their location on the sarcolemma [47, 57]. The DGC-bound α/β-syntrophin and α-dystrobrevins 1 and 2 can directly interact [57]. α-Dystrobrevin also binds to intermediate filament proteins desmuslin and syncoilin [58, 59] and associates with dystrophin increasing its affinity to the DGC [60]. Furthermore, Dp71 containing both CR and CT domains was shown to directly interact with myospryn, a muscle-specific protein kinase A (PKA) anchoring protein [61]. It is important to note that the size of the CT domain varies in Dp140, Dp116, and Dp71, dependent on alternative splicing of exons 71–74 and 78 [25–27], and even though this domain is not required for the assembly of the DGC, its length may regulate the syntrophin and dystrobrevin isoform composition [28].

**Table 1:** Amino-acid and structural comparison of distinct protein binding domains and motifs in dystrophin and utrophin

Binding site	Dystrophin amino acids (NP_003997.2)	References	Utrophin amino acids (NP_009055)	Amino acid identity (%)	Amino acid similarity (%)	TM score
ABD1	9–246	[224, 225]	28–261	73	87	0.94848
MTBS1	710–1965	[226]	687–1965	38	60	0.66477
MK2BS	1155–1367	[40]	1125–1334	55	73	0.90185
ABD2	1416–1880	[36]	1383–1855	42	67	0.47078
nNOS/STBS	1992–2208	[41]	1849–2080	40	62	0.95623
MTBS2	2471–3040	[226]	2229–2797	46	69	0.18199
DGBS	3054–3446	[227]	2811–3203	80	88	0.77853
STBS	3444–3535	[228]	3201–3311	66	81	0.41259
α-STBS	3444–3494	[228]	3201–3251	63	80	0.43252
β-STBS	3495–3535	[228]	3265–3292	68	82	0.28808
α-DBBS	3501–3541	[229]	3265–3293	69	82	0.63588
WW	3055–3092	[50]	2812–2849	84	94	0.85983
EF-hands	3130–3206	[50]	2892–2963	72	81	0.90849
ZZ	3307–3354	[230]	3064–3111	92	97	0.82243

*ABD1/2* actin-binding domain 1/2, *MTBS1/2* microtubules binding site 1/2, *MK2BS* Ser/Thr kinase MAP/microtubule affinity-regulating kinase 2 (MARK2) binding site, *nNOS* neuronal nitric oxide synthase, *DGBS* β-dystroglycan binding site *α/β-STBS*, syntrophin binding site, *DBBS* α-dystrobrevin binding site, *EF-hands* motif composed of two EF-hands 1 and 2, *WW* WW domain, *ZZ* ZZ-type zinc finger motif

The dystrophin R20–R23 of MTBS2 was extended to R20–R24 allowing the analysis of the full region between two hinges, H3 and H4.

The stability of the dystrophin interaction with the sarcolemma can be enhanced by its direct interaction with phospholipids through several domains, including the CT domain, the CR domain, and H4, as well as spectrin-like repeats R1–R3 and R10–R12 (Fig. 1B). In the latter case, the study by Zhao et al. showed that following rAAV-mediated gene transfer, the GFP–R10–R12 fusion protein was detectable in both the cytoplasm and the sarcolemma of *mdx* mice and dystrophic dog muscles, while only in the cytoplasm of cardiomyocytes from *mdx* mice [62]. In agreement, a single lipid-binding domain was recently identified within the ABD2 in the C-terminal end of dystrophin R12, indicating a potential F-actin/dystrophin/membrane lipids ternary complex in skeletal muscle cells [36]. As these results were obtained using separate protein domains, it would be interesting to test whether ABD2 retains partial sarcolemmal localization in vivo when bound to the remaining dystrophin. This is particularly interesting in the context of a study showing that human dystrophin structurally changes upon binding to anionic membrane lipids, suggesting that the interaction properties of a small fragment can differ from the whole protein [63].

## Utrophin and Its Sequential and Structural Comparison to Dystrophin

So far, five full-length utrophin (395 kDa) first exons have been identified in humans (A, A’, B, B’, and F) (Figs. 1A and 2A) [64]. Activation of their corresponding promoters results in mRNA transcripts that have different expression patterns [64]. While Up395a and Up395b have been studied for years, Up395f protein with a unique N-terminus was detected in adult tissues more recently [64–66]. Ubiquitously expressed at the sarcolemma in embryonic muscle, Up395a is also present in adult muscle, notably at NMJs, myotendinous junctions (MTJs), and at the sarcolemma of regenerating myofibrils, as well as in other tissues, including choroid plexus in the brain, pia mater, and renal glomerulus [66]. In contrast, Up395b localization is limited to endothelial cells and blood vessels, whereas immunofluorescence microscopy analyses revealed Up395f in regenerating fibers, perivasculature, and in the interstitial endomysium of mouse muscles [64, 66]. *UTRN-A* and *-F* also have higher expression in slower than in fast fibers, indicative of a specific mechanism dependent on a muscle fiber type that impacts their expression [64]. As in the case of dystrophin, utrophin is also transcribed from promoters that give rise to shorter isoforms (Up140, Up113, Up109, Up71) (Fig. 1A) [66, 67].

The full-length utrophin and dystrophin are involved in formation of protein complexes connecting the extracellular matrix with the cytoskeleton inside the cell and the UGC (Fig. 1B), similarly to the DGC, was shown to protect efficiently the sarcolemma against the contraction-induced damage, especially in nonexercised mice [45, 68, 69]. As in the case of dystrophin, utrophin consists of four major domains (Fig. 1): the NT/ABD1 domain that binds F- and γ-actin, the CenR domain composed of spectrin repeats, the CR domain, and the CT domain [66, 70]. Importantly, utrophin similarly to dystrophin has the capacity to bind β-dystroglycan, α-dystrobrevin-1, plectin, and ankyrins [19, 24, 53, 60], although the utrophin affinity for β-dystroglycan might be lower than that of dystrophin [71]. Despite the structural similarities, the CenR of Up395 is composed of 22 spectrin repeats, in contrast to the 24 repeats in Dp427, and the full-length utrophin lacks the sequence corresponding to the spectrin repeats 15 and 19 of dystrophin, and thus the ability to interact with nNOS and microtubules are compromised [45, 49]. Importantly, the lack of efficient locating of nNOS and microtubules to the complex impairs the regulation of the blood flow to the muscles [45, 72] and contributes to the contraction-induced myofiber damage [49], respectively. Furthermore, while dystrophin binds actin through ABD1 and ABD2, utrophin lacks the functional ABD2 [73] and its interaction with actin occurs through ABD1 that is additionally influenced by spectrin repeats R1–R10 [33] and by the 28 amino acid extension at the N-terminus [74]. In vitro experiments revealed also that mechanical properties of dystrophin and utrophin might differ in myofibers. Specifically, phosphorylation of utrophin increases its stiffness, predisposing it to function at MTJs (where it naturally occurs) rather than at the sarcolemma as a molecular shock absorber [75, 76].

To further explain the differential binding capacity of dystrophin and utrophin, we compared their nucleotide and amino acid sequences. The analysis showed that two main muscle isoforms, Dp427m and Up395a, share 86.37% nucleotide (NG_012232.1, NG_042293.1) and 41.61% amino acid (NP_003997.2, NP_009055.2) sequence identity. The extended amino acid comparison revealed that depending on the domain, the amino acid identity may rise to 81% (Table 1). Particularly, the highest homology was found in regions that enable both dystrophin and utrophin interaction with β-dystroglycan via the DGBS (amino acid 81% identity and 88% similarity), F-actin via ABD1 (73% amino acid identity and 87% similarity), α-dystrobrevins via DBBS (69% amino acid identity and 82% similarity), and α/β syntrophins via the STBS (66% amino acid identity and 81% similarity). In contrast, the sequence analysis revealed that the dystrophin region responsible for nNOS/α-syntrophin binding (nNOS/α-syntrophin binding site, nNOS/STBS) differs from that of utrophin, with only 40% identical and 62% similar amino acids.

We further compared the dystrophin MARK2 binding site (MK2BS), the second domain with actin-binding properties (ABD2), and the crucial region shown to bind to and organize microtubules (R20–R23) with utrophin corresponding amino acid sequences. For MK2BS, the matching sequence in utrophin was found to encompass R8–R9, which is the same region that was detected by Yamashita and team [40], with 55% identical and 73% similar amino acids. Of note, *mdx* mice exhibit significant loss of MARK2 in germ cells during spermatogenesis, while *mdx*/*utrn*^+/−^ mice show an even more significant decrease of MARK2, leading to apoptosis and decreased proliferation of spermatogenic cells [77]. These results suggest that the amino acid similarity in the MK2BS at the level of 73% suffices for dystrophin and utrophin interaction with MARK2 and that utrophin, like dystrophin, might regulate stem cell division [39]. Previous results showed that utrophin differs from dystrophin in its ability to bind and organize microtubules and does not contain ABD2 [49, 73]. Our analysis revealed the highest homology to dystrophin ABD2 domain in utrophin in a region between the end of the 10th spectrin repeat and the 15th spectrin repeat (42% amino acid identity and 67 % similarity), to MTBS1 between the 4th and the 15th spectrin repeat (38% amino acid identity and 60% similarity), and MTBS2 between the 18th and the 21st spectrin repeat (46% amino acid identity and 69% similarity). These values are below the results obtained for MARK2 while on par with the nNOS homology comparison values, thus they indicate that alterations in the amino acid sequence are responsible for the observed changes in the interaction with actin filaments and microtubules for dystrophin and utrophin [49, 73]. In summary, the sequence analysis showed that the loss of protein domain homology corresponds well with functional studies showing differential binding properties of dystrophin and utrophin.

**Fig. 3:**
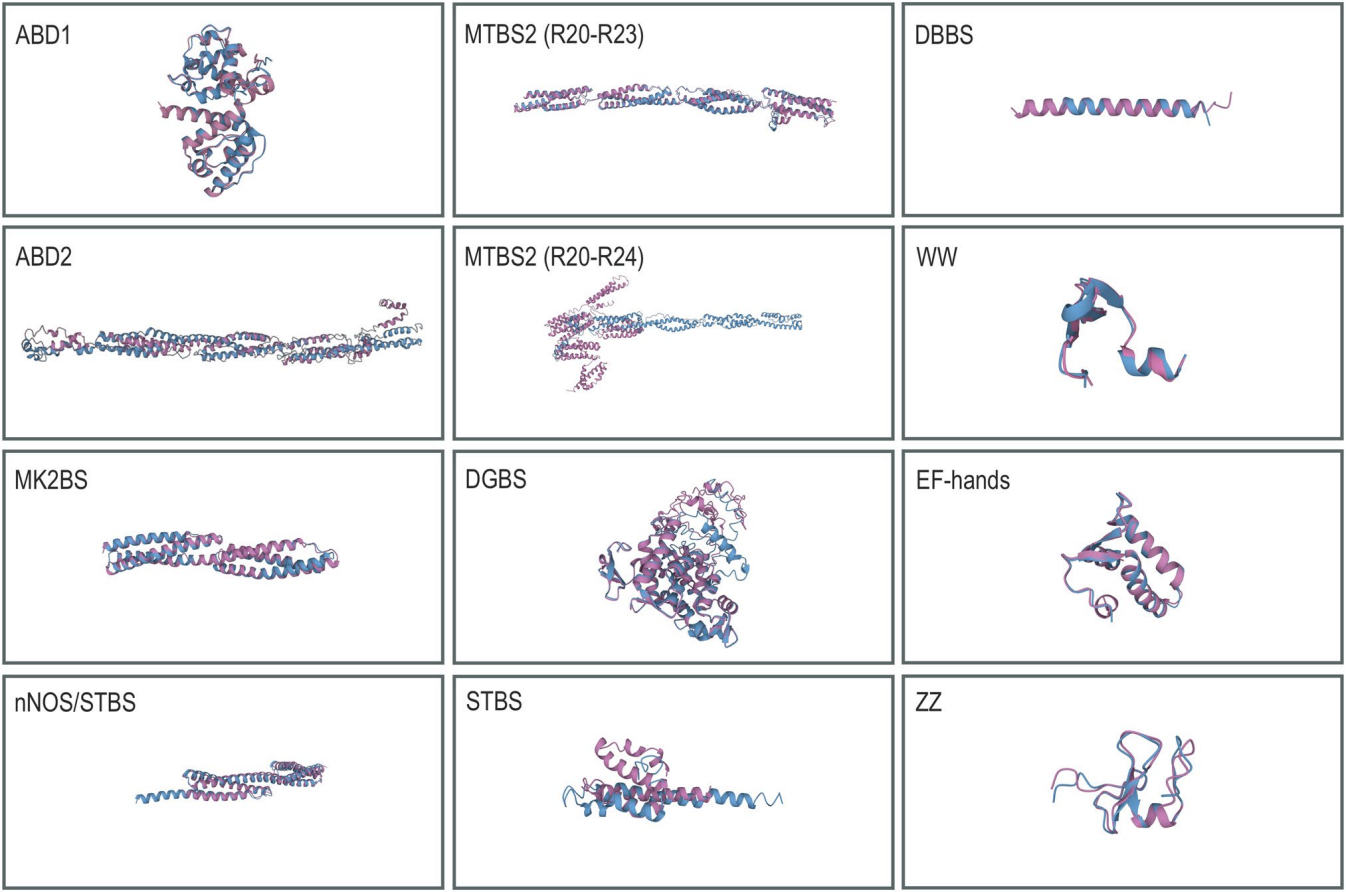
Comparison of 3D structures of protein binding motifs of dystrophin and utrophin. The images represent superimposed 3D structures (TM-align software) of distinct domains and binding sites of dystrophin (pink) and utrophin (blue). The microtubules binding site 2 (MTBS2) is shown as the repeat sequence R20–R23 (the sequence that directly binds microtubules) and R20–R24 (includes the whole region between two hinges). *ABD1/2* actin biding domain 1/2, *DBBS* α-dystrobrevin binding site, *DGBS* β-dystroglycan binding site, *EF-hands* a region composed of two EF-hands motifs, 1 and 2, *MK2BS* Ser/Thr kinase MAP/Microtubule affinity-regulating kinase 2 (MARK2) binding site, *nNOS/STBS*, nNOS/α-syntrophin binding site, *MTBS1/2* microtubules binding site 1/2, *STBS*, α/β-syntrophins binding site, *WW* WW motif, *ZZ* ZZ-type zinc finger motif

**Fig. 4:**
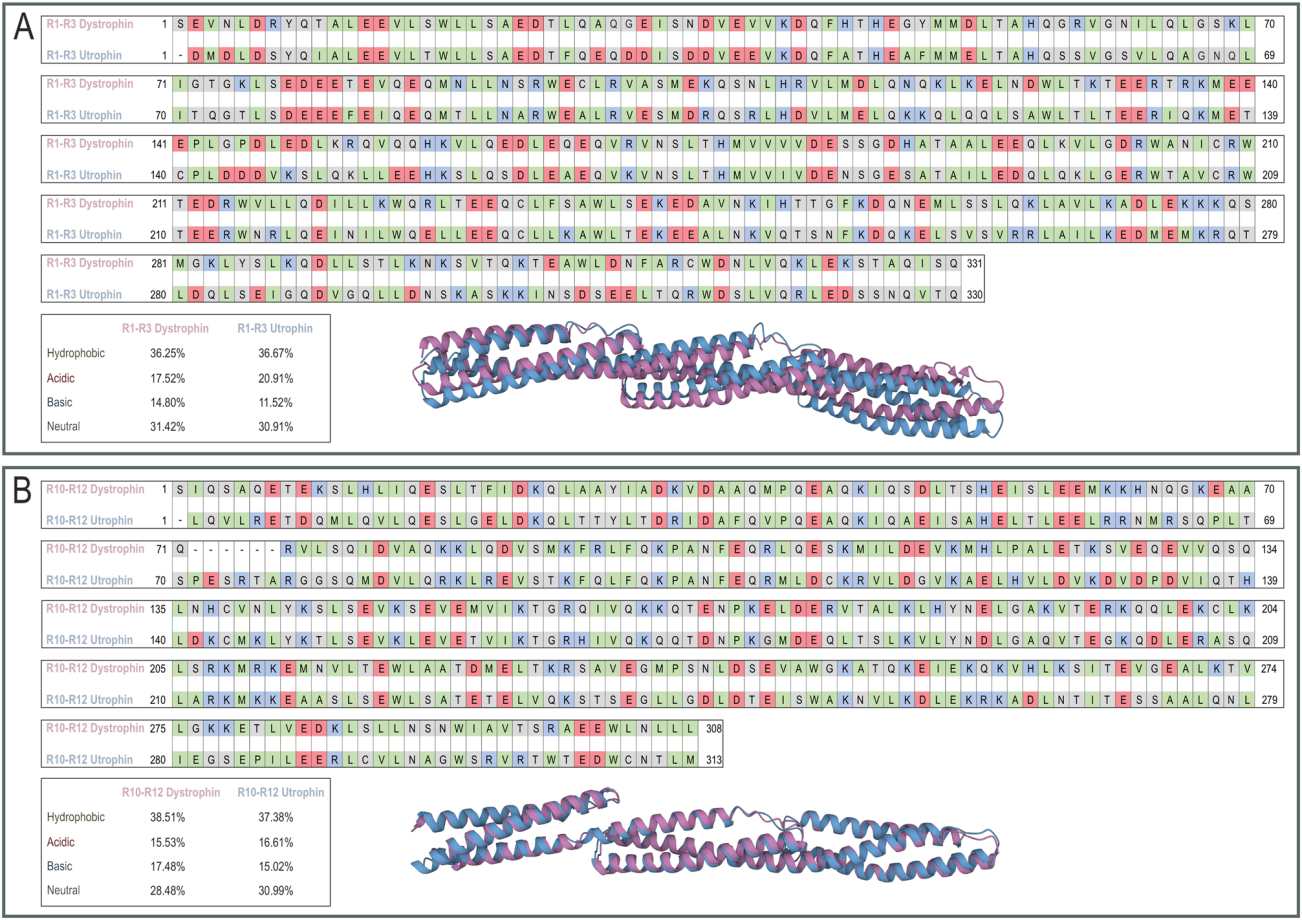
Comparison of dystrophin and utrophin spectrin repeats R1–R3 and R10–R12. Amino acid alignments and 3D structures of R1–R3 (**A**) and R10–R12 (**B**) of dystrophin and utrophin are shown. Green indicates hydrophobic amino acids (A, alanine; F, phenylalanine; I, isoleucine; L, leucine; M, methionine; P, proline; V, valine; W, tryptophan); red, acidic amino acids (D, aspartic acid; E, glutamic acid); blue, basic amino acids (H, histidine; K, lysine; R, arginine); gray, other amino acids (C, cysteine; G, glycine; N, asparagine; S, serine; T, threonine; Q, glutamine; Y, tyrosine). The superimposed 3D structures of spectrin repeats R1–R3 and R10–R12 of dystrophin (pink) and utrophin (blue) were generated with the TM-align software

To further investigate similarity and differences between dystrophin and utrophin, an in silico folding analysis of various domains was performed (Figs. 2B, 3, and 4, Table 1). We created 3D models for domains of interest using the I-TASSER tool [78] followed by comparison of structures based on a quantitative assessment of protein structure similarity (TM score) that is determined by the TM-align algorithm [79]. The TM score has values between 0 and 1, where scores below 0.17 correspond to unrelated proteins, 0.5 to proteins having generally the same folding characteristics, and 1 indicating a perfect match. In silico in-between comparison of dystrophin and utrophin revealed that regions showing high amino acid similarity, including ABD1 (87%), MK2BS (73%), DGBS (88%), and DBBS (82%), also show high structural resemblance (0.95, 0.90, 0.78, 0.64 TM scores, respectively) (Table 1, Fig. 2B). Analogously relatively high structural resemblance was detected within DGBS regions, spanning the WW domain, EF hands, and the ZZ motif (0.86, 0.91, 0.82 TM scores, respectively) (Table 1). This data thus overlaps with scientific findings showing that utrophin is able to retain β-dystroglycan [80], actin [81], α-dystrobrevin [82], and MARK2 [40] binding properties of dystrophin. However, to our surprise, the STBS, despite the 66% amino acid identity, exhibited relatively low structural similarity (0.41 TM score), with particularly low resemblance in the β-STBS (0.29 TM score).

As dystrophin improves its stability and binding to the sarcolemma through spectrin repeats R1–R3 (Dp427m, 337–667 aa; Up395a, 308–637 aa) and possibly R10–R12 (Dp427m, 1368–1676 aa; Up395A, 1336–1648 aa) [62], we also assessed the sequence homology in the corresponding utrophin regions. The analysis showed that paralogous subdomains in utrophin display average (on par with MK2BS) amino acid identity (56% and 49%) and similarity (77% and 73%) and high structural resemblance (0.73589 and 0.96328 TM score) to dystrophin R1–R3 and R10–R12 regions, respectively (Fig. 4). We also compared the content of nonpolar, polar uncharged, and charged amino acids in dystrophin and utrophin. Importantly, the analysis showed that the percentages of corresponding amino acid groups in R1–R3 and R10–R12 regions of dystrophin and utrophin are alike (Fig. 4). To conclude, these results indicate that utrophin, similarly to dystrophin, might directly interact with sarcolemma; however, further experimental analyses are required to confirm this hypothesis.

## Replacement of the Mutated *DMD* Gene

A defective *DMD* gene can be replaced with a dystrophin or utrophin coding sequence delivered by rAAV vectors [83]. An important aspect of rAAV vectors is their ability to administer new genes to the skeletal and cardiac muscles of adult mammals in a systemic manner [84]. Other advantages of rAAV vectors include relatively long and stable gene expression and the ability to infect both dividing and nondividing cells [85, 86]. Moreover, AAV DNA functions as an episome in the cell, rarely integrating into the host’s genetic material, which significantly reduces risks associated with mutagenesis [87].

The downside of rAAV vectors is their small size, which translates into the possibility of accepting a limited amount of DNA, which is approximately 5 kbp [88]. This is especially important in the case of the coding sequences of the full length *DMD*/*UTRN* mRNAs, as they are above 10 kb. A solution to this problem emerged when one of the BMD patients had mild disease symptoms, walking on his own at 61 years of age, despite the extensive deletion of about 46% in the *DMD* gene [89]. Based on this discovery, several differently structured micro- and mini-dystrophins (μ/mDys) have been proposed, from which fragments of the gene coding sequence have been deleted [90]. The mDys gene, similar to the *DMD* coding sequence present in BMD patients, is approximately 6–8 kbp in size and thus its delivery requires the use of two vectors [91]. In contrast, smaller versions of the *DMD* coding sequence (μDys) can fit seamlessly into rAAVs [12]. Importantly, the strategy grounded on rAAV vector delivery could be applied to all DMD and BMD patients, without the knowledge of a specific mutation type.

AAVs come in the form of various serotypes that differ in tissue tropism and transduction efficiency that may also be species dependent [92]. In striated muscles, AAV9 and AAVrh74 (highly similar to AAV8) are currently used in clinical trials for DMD [13, 93, 94] and an AAV9 mutant, AAVMYO, showed very high transduction efficiency in more recent studies [95]. Some rAAV vectors have also been shown to be able to edit satellite cells, albeit with reduced efficiency [85, 86]. This may indicate the need for repeated rAAV vector delivery to maintain the therapeutic effect of μ/mDys. However, it is important to note that each delivery following the initial rAAV vector administration would require immunosuppression to prevent the body’s response against the viral capsid proteins acquired after the first injection [93, 96]. The time at which therapeutic rAAV vectors should be delivered again is not fully predictable but only estimated to reflect the half-life of adult fibrils. Studies in large animals have shown that this period can be 5–15 years or longer [92].

**Fig. 5:**
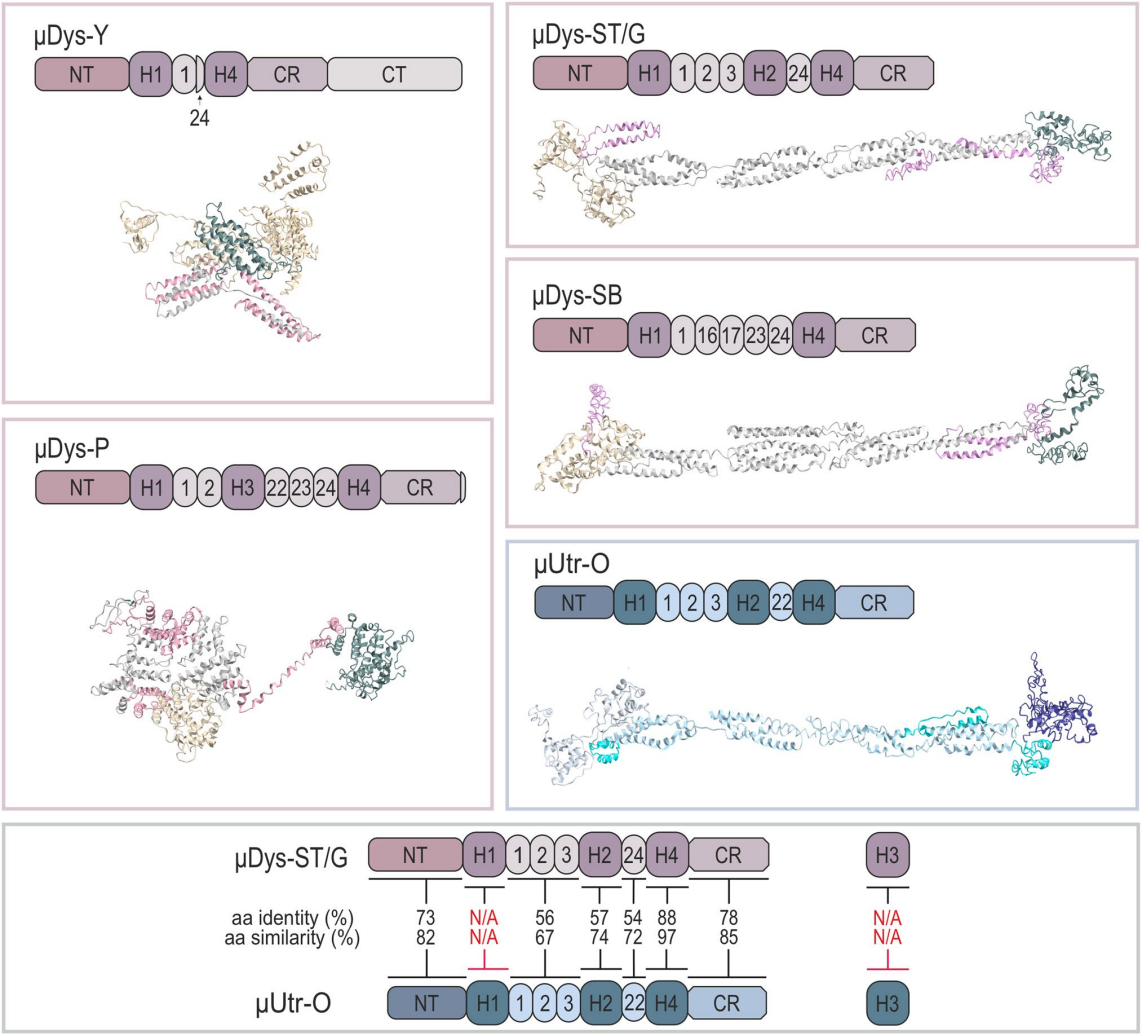
Graphical representation, 3D structures, and sequence alignment of μDys and μUtr proteins. 3D structures were predicted with the I-TASSER software. Note that μDys–Y and μDys–P show a more condensed structure, while μDys–ST/G, μDys–SB, and μUtr–O are linear, being more comparable to the full-length dystrophin. Sequence alignment of μDys and μUtr proteins revealed that H1 and H3 regions are dissimilar (lower panel, marked red). *μDys–Y* micro-dystrophin from [118], *μDys–P* micro-dystrophin manufactured and tested by Pfizer [102], *μDys–ST/G* micro-dystrophins manufactured and tested by Sarepta Therapeutics (μDys–ST) and by Genethon and Sarepta Therapeutics (μDys–G) [90], *μDys–SB* micro-dystrophin manufactured and tested by Solid Biosciences [113], *μUtr–O* micro-utrophin designed by Odom et al. [112, 187]. *N/A* not applicable

Observation of the severity of the disease phenotype in patients with various mutations allowed to determine key areas of the *DMD* gene to preserve the greatest functionality of the resulting protein [88, 97]. Both the actin-binding N-terminal and the β-dystroglycan-binding domains are considered essential [1], with restoration of the DGC alone shown to be insufficient to prevent fiber degeneration [98–100]. By contrast, the CT domain as well as the whole central portion of dystrophin is usually omitted from μDys. Clinical trials have commenced on several proteins. Currently, four rAAV vectors [101] carrying three different μDys are in human trials: (1) manufactured and tested by Pfizer (μDys-P) [102–104], (2) Sarepta Therapeutics or Genethon in collaboration with Sarepta Therapeutics (μDys–ST and μDys–G, respectively) [90, 105–112], and (3) Solid Biosciences (μDys–SB) [113–115] (Fig. 5, Table 2). Sarepta’s studies involve the use of rAAV–rh74-containing MHCK7 promoter (μDys–ST) or AAV-8 with Spc5.12 (collaboration with Genethon, μDys–G) while others introduce rAAV9 with a muscle-specific (μDys–P) and CK8 (μDys–SB) promoters, respectively. Preclinical experimental data indicate that all rAAV–μDys vectors used in clinical trials allow for expression of truncated dystrophin in skeletal muscles of dystrophic mice [90, 102, 105–109, 113–116] and dogs [111], leading to pathology reduction and fiber size normalization (Table 2). Importantly, μDys–ST (Elevidys) has been recently approved by the FDA through the accelerated approval pathway for the treatment of DMD boys ages 4–5 years based on the preliminary clinical data [117]. Each of the tested μDys has a functional actin and β-dystroglycan binding sites; however, different overall sequence and structure that might determine their ability to compensate for the full-length dystrophin absent in DMD patients. μDys–SB stands out from the other truncated dystrophins as it contains the nNOS binding site (Table 2).

**Table 2:** Comparison of effectiveness of selected μDys and μUtr in in vivo testing

	Vector (injection mode)	Animal models/human studies	μDys/μUtr: sk. muscle/heart	β-DB/α/β-ST/SG/	nNOS	CNFs/muscle force	Fiber size normalization/body weight	Immune response against μDys/μUtr	References
μDys–Y	E1–Ad (i.m.)	*mdx*	+ + +/N/A	+ + +/+ + +/+ + +/	N/A	N/A/N/A	N/A/N/A	N/A	[118]
μDys–P	rAAV (i.m.) rAAV2.5 (i.m.)	*mdx*, humans	+ + +/N/A	NA/NA/+ + +/	N/A	+ + +/N/A	+ + +/N/A	Immune response (humans)	[102–104]
μDys–ST/G	rAAV2 (i.m.) rAAV5 (i.m.) rAAV6 (i.v.) rAAV9 (i.m.) rAAV9 (i.v.) rAAV2/8 (i.v.)	*mdx*, *mdx*/*utrn*^-/-^/ GRMD, GSHPMD, humans	+ + +/+ + +	+ + +/+ + +/+ + +/	−	+ + +/+ + +	+ + +/− (mdx)	Immune response (*mdx*, GSHPMD, humans), transient immune response (GRMD)	[90, 105–107, 110–112, 123, 231]
μDys–SB	rAAV9 (i.v.) rAAV6 (i.m.) rAAV6 (i.p.)	DBA/2J-*mdx*, *mdx*^4cv^, *mdx*	+ + +/+ + +	+ + +/+ + +/+ + +/	+ + +	+/+ + +	+ +/+	N/A	[113, 114]
μUtr–O	rAAV9 (i.p.) rAAV9 (i.m.) rAAV9 (i.v.)	*mdx*	+ + +/+ + +	+ + +/+ + +/+ + +/	N/A	+ + +/+ + +	+++/N/A	No immune response	[107, 112, 187]

*μDys–Y* micro-dystrophin [118], *μDys–P* micro-dystrophin manufactured and tested by Pfizer [102], *μDys–ST/G* micro-dystrophin manufactured and tested by Sarepta Therapeutics and by Genethon and Sarepta Therapeutics [90], *μDys–SB* micro-dystrophin manufactured and tested by Solid Biosciences [113], *μUtr–O* micro-utrophin [187], *i.v.* intravenous, *i.m.* intramuscular, *i.p.* intraperitoneal, + + + completely normalized (compared to WT), + + greatly improved (compared with *mdx*), + improved (compared with *mdx*), – negative effect, *N/A* not applicable, Muscle force includes contractile force and distance on treadmill analyses, *GRMD* golden retriever muscular dystrophy dog, *GSHPMD* German shorthaired pointer muscular dystrophy dog

Based on the available studies and in silico structure analysis, we compared all three μDys that are currently in clinical trials and additionally with the first μDys (μDys–Y) designed by Yuasa et al. [90, 102, 113, 118]. Both μDys–SB and μDys–ST/G showed similar rod domain structure to the one present in dystrophin, while μDys–P and μDys–Y revealed a more condensed most probable structure, with the nonpresent spectrin-like repeat “linear folding” (Fig. 5). However, it needs to be taken into consideration that the I-TASSER tool shows a few proposed models and a structure (although less probable) similar to the μDys–SB and μDys–ST/G structures were also obtained. The diversity in μDys–P and μDys–Y models suggests nonetheless that these μDys proteins might have problems with suitable folding.

Despite the many benefits of rAAV-based therapy, there are still some aspects that will need to be further investigated and resolved. Since rAAV vectors exist in the cell as episomes, they will be lost during cell division, and the level of dystrophin will decrease [85, 119]. Another problem is the immune response induced by AAV [88, 120]. Some patients have pre-existing antibodies against specific serotypes and even if they are seronegative, the first gene transfer might exclude them from rAAV vector re-administration. Theoretically, this could be overcome by using other rAAV serotypes. Some researchers are also considering the use of plasmapheresis or other methods to weaken the body’s immune response to the vector [121]. The immune response can also be activated by the newly generated dystrophin [103, 107, 112, 122, 123]. In people with DMD, the expression of the gene does not usually lead to the production of a functional protein. Therefore, when a vector encoding the m/μDys gene is introduced into the body, the resulting protein might be recognized as foreign. In response, the body might start producing T lymphocytes, significantly reducing the effectiveness of the therapy as well as posing threats to the patient. One of the proposed solutions is the use of an additional vector that would reduce the immune response and lead to a more efficient expression of the delivered gene [124]. Other emerging strategies rely on the use of transient immune suppression to prevent activation of B and T cells, leading to the recognition of dystrophin or AAV proteins by effector cells [92].

In contrast to dystrophin constructs, the use of the utrophin sequence allows the immune response to be bypassed and the therapy to work effectively [107, 112] (Table 2). Particularly, 2 weeks following the intramuscular delivery of rAAV–μUtr and rAAV–μDys to German shorthaired pointer muscular dystrophy (GSHPMD) dogs, in which a deletion of the whole *DMD* gene occurred [125], Song et al. observed a strong immune system response against the μDys, with no adverse reactions to μUtr [112]. Similarly, recent experiments in mice showed that μUtr induces lower overall immunogenicity than μDys [107] and that delivery of rAAV carrying μDys gene induces generation of dystrophin-specific antibodies [122]. Also, the first results from the ongoing clinical trials showed that μDys-ST/G can induce serious adverse effects associated with anti-dystrophin T-cell responses in patients lacking N-terminal epitopes [123]. In agreement, our sequence alignment analyses revealed that while μDys–ST/G and μUtr–O show relatively high amino acid identity and similarity (61% and 76%, respectively), H1 has no significant similarity between both proteins and is the least conserved region (Fig. 5). Additionally, the hinge 3 region, which is present in μDys–P also reveals no significant similarity between dystrophin and utrophin (Fig. 5). These results indicate that H1 and H3 in μDys could be sequentially optimized for synthesis in DMD patients based on the utrophin sequence. On the other hand, μUtr might be generally a better solution to dystrophin-based therapies due to the “foreign” properties of μDys.

## Exon Skipping with AONs

**Fig. 6:**
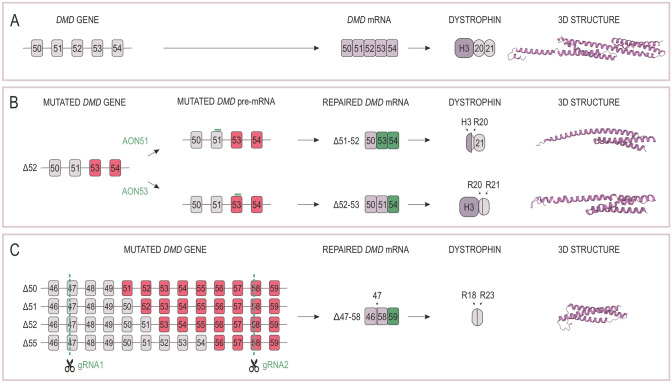
Restorative repair of the *DMD* gene expression with antisense oligonucleotides (AONs) and CRISPR/Cas9 technology. **A** Expression of the *DMD* gene in control samples based on a *DMD* fragment that encompasses exons 50–54 that is transcribed and translated into a protein region composed of H3 and spectrin repeats R20 and R21. **B**, **C** Deletion of *DMD* exons 50, 51, 52, or 55 (Δ50, Δ51, Δ52, Δ55) causes DMD as it changes the dystrophin reading frame and the protein cannot be synthesized. In (**B**) is shown an example where the reading frame can be restored in patients carrying Δ52 mutation via the use of AONs that induce skipping of exons 51 and 53 in pre-mRNA. Note that although the truncated dystrophin is missing part of hinge 3 (H3) and R20 or part of R20 and R21, the synthesized protein fragments have largely unaffected 3D structures. As presented in **C**, the dystrophin reading frame in patients with distinct mutations, including Δ50, Δ51, Δ52, Δ55, can be restored by deleting a relatively large fragment of the *DMD* gene with the CRISPR/Cas9 technology. Note that the gRNAs are designed to cut within exons 47 and 58 to remove a relatively large region within the rod domain (Δ47–58) so that the perfect spectrin repeat structure is recreated from the remaining R18 and R23 fragments [161]

The pre-mRNA exon skipping approach using RNA or DNA antisense oligonucleotides (AONs) is grounded on the fact that most DMD patients can theoretically produce dystrophin similar to that produced in BMD patients if the reading frame is corrected. AONs can be synthesized based on a variety of chemical backbones [126], with commonly used 2′O-methyl-ribo-oligonucleoside-phosphorothioate (2′OMePS) and phosphorodiamidate morpholino oligomers (PMO) [127, 128]. Upon their delivery to the cell and binding to specific sequences in the *DMD* pre-mRNA, the mutant exon and sometimes additional contiguous exons can be skipped during mRNA maturation. Ultimately, this process can restore the reading frame and result in production of a partially functional dystrophin protein (Fig. 6A, B). Most DMD patients have deletions of one or more exons, which are usually grouped in two hot spot regions, the first region spanning exons 3–9 and the second encompassing exons 45–55 [129]. Mutations in these fragments are observed in 7% and 47% of all DMD patients, respectively [130]. It is estimated that approximately 70% of DMD patients who have a deletion could be treated by skipping one exon [131]. Multiple exons skipping has emerged as an alternative method that could extend therapeutic application of this approach, in which case, administration of a cocktail of multiple antisense oligonucleotides results in skipping of multiple exons to restore the dystrophin mRNA open reading frame [132].

Independently of each other, two types of antisense oligonucleotides have been developed for exon 51 skipping in patients, based on a PMO (eteplirsen) and 2′OMePS (drisapersen) modification [133–135]. In contrast to drisapersen (Kyndrisa, BioMarin Pharmaceutical), eteplirsen (Exondys, Sarepta Therapeutics) received accelerated approval in 2016 by the FDA based on a slight increase in dystrophin levels, 0.4% and 0.9% of the control level, in 13 and 11 DMD patients after 48 and 180 weeks of treatment, respectively. Eteplirsen also slowed progression of the disease, as measured by the distance achieved by the 6-minute walk distance (6MWD), when patients treated for 3 years were compared with a historical control group of patients with DMD who had only received supportive care. However, FDA noted that no functional benefit of eteplirsen treatment has yet been convincingly demonstrated and manufacturers have been asked to submit confirmatory data. Despite approval from FDA and still ongoing tests, EMA decided to refuse the marketing authorization of eteplirsen in 2018 (EMA/621972/2018). Sarepta Therapeutics received FDA approval also for PMOs targeting exons 45 and 53 with active substances known as casimersen (Amonydys 45) [136, 137] and golodirsen (Vyondys 53) [138], respectively, which together with eteplirsen are under phase 3 of clinical trial [casimersen and golodirsen (NCT02500381), eteplirsen (NCT03992430)]. Additionally, NS Pharma received approval in 2020 in Japan and the USA for a PMO designed to skip exon 53 (viltolarsen (Viltepso, NS-065/NCNP-01) [139, 140]. Both Viltepso and yet another PMO, brogidirsen that targets exon 44 (NS-089/NCNP-02) are currently in clinical trials (NCT04337112, NCT04129294).

The limitations of the early AON exon-skipping strategies include low myocardial efficacy, poor cellular uptake, and rapid exit from the circulatory system. More recent approaches tackle some of these drawbacks. These include delivery of SQY51, a tricyclo-DNA molecule targeting exon 51 (NCT05753462), developed by SQY Therapeutics [141] and AOC 1044 from Avidity Biosciences (NCT05670730). The latter one induces exon 44 skipping and is bound to a monoclonal antibody for enhanced uptake to muscle cells. Published data on systemic delivery of the tricyclo-DNA AON to *mdx* mice are particularly encouraging, as high-level exon 51 skipping was observed not only in skeletal muscles but also in the heart and, to a lesser extent, in the brain [141]. Since both AONs and dystrophin transcripts are not permanent, treatment with AONs is not a one-time procedure and must be repeated [142]. Importantly, while approximately 20% of uniformly distributed endogenous dystrophin in myofibers and cardiomyocytes may suffice to halt progression of DMD [143], much lower levels might be already beneficial for patients [144, 145].

## Stop Codon Readthrough

Approximately 10–15% of DMD cases are due to nonsense mutations in the dystrophin gene [146]. Blocking a premature stop codon could be achieved by suppressing the reading of premature stop codons by binding therapeutic molecules to ribosomes, which enable the full-length translation of the modified dystrophin protein [147]. One such molecule is Ataluren (PTC124), also known as Translarna [148]. Treatment of *mdx* mice rescued striated muscle function within 2–8 weeks of drug administration, with approximately 20–25% dystrophin levels of the amount detected in the muscles of healthy control mice [149]. Furthermore, the treatment resulted in a significant reduction in creatine kinase levels and dystrophin presence in the heart of *mdx* mice. These promising results led to first clinical trials in healthy volunteers and DMD patients, showing high tolerability and safety when administering the drug as well as a positive effect on the 6MWD result of the affected boys [150–152]. Based on these results, EMA approved ataluren in 2014 for treatment of DMD caused by nonsense mutations in patients aged 5 years and over [153] (EMEA/H/C/002720). In contrast, the FDA did not agree to use it in the USA for this purpose due to insufficient data showing a positive effect of the treatment (NCT00759876). Additional research is underway to demonstrate the positive effect of such therapy [148].

## Gene Editing with CRISPR/Cas9

CRISPR/Cas9 applications were confirmed to have a positive outcome in DMD disease models, including those based on a point mutation correction or in-frame deletion of premature stop codon [154, 155], exon or exons deletion [155–164], exon in-frame deletion (reframing) [155, 159, 165], exon knock-in [159, 166], or base editing [167, 168]. CRISPR technology was introduced to the *DMD* gene editing through a nonsense point mutation correction in 2014 [154]. Long et al. used SpCas9 and a single sgRNA, showing the ability of CRISPR/Cas9 to correct the sequence in *mdx* embryos. The genetically mosaic animals contained 2–100% correction of the *DMD* gene, which contributed to decreased values of creatine kinase in serum as well as enhanced muscle performance when compared with dystrophic *mdx* mice. The dystrophin open reading frame (ORF) was also restored, e.g., in myoblasts and induced pluripotent stem cells derived from DMD patients by a single sgRNA pair inducing deletion of exons 45–55 [158, 169]. In contrast to the AON-based therapy, the CRISPR/Cas9 can be designed to precisely cut within exons to restore not only the ORF but also the structural properties of spectrin repeats. Specifically, Duchene et al. demonstrated that SaCas9 administration with two sgRNAs to a humanized mouse model of DMD (del52h*DMD*/*mdx*) can lead to DNA breaks in exons 47 and 58, restoration of the ORF, and synthesis of a functional dystrophin with normally phased spectrin-like repeats [161] (Fig. 6C). Interestingly, the highest repair outcome, at the level of 86%, was observed in the hearts of dystrophic animals, as has been seen with deletion strategies [170].

CRISPR/Cas9 technology also allows for larger insertions and Pickar-Oliver et al. showed that the full-length dystrophin can be obtained in del52h*DMD*/*mdx* mice based on the homology-independent target integration approach (HITI) following dual rAAV vector delivery of CRISPR/Cas9 and a donor DNA sequence [166]. To increase availability of the treatment to a higher number of patients, a larger fragment was additionally delivered, containing not only exon 52 but also exons up to 79, along with a polyA sequence. After administration of the 28-exon sequence, the mice achieved correction in the hearts up to 7% at the DNA and over 25% at the mRNA level, while much lower efficiencies were obtained in skeletal muscles [166]. Notably, this level of dystrophin synthesis might suffice to improve the cardiac muscle function [45, 171].

**Fig. 7:**
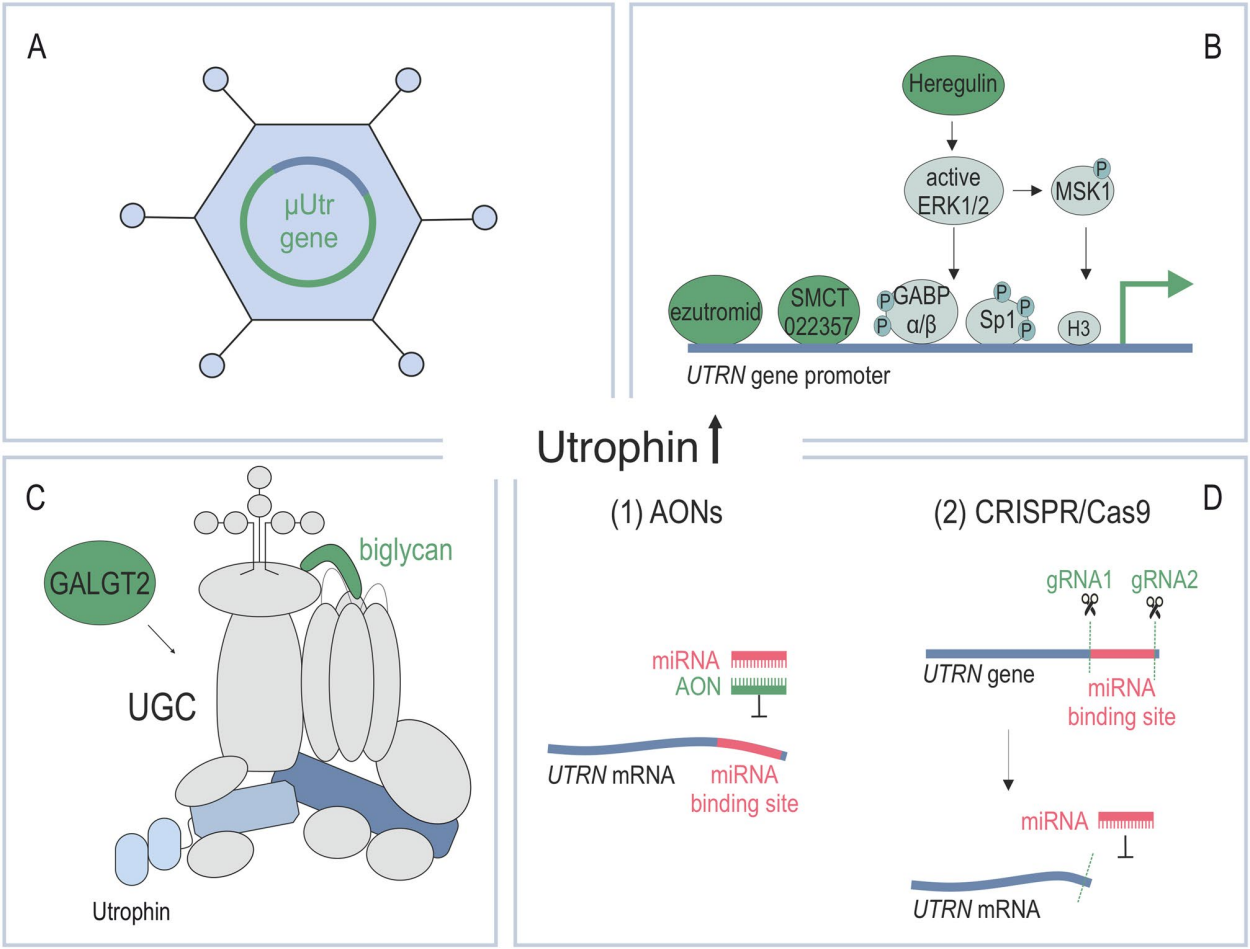
Schematic representation of utrophin-based therapies for DMD. Utrophin increased levels could be achieved through rAAV-mediated delivery of genes encoding μUtr (**A**), activation of the endogenous *UTRN* gene promoter directly through ezutromid/SMT022357, or indirectly via heregulin that induces distinct signaling events (**B**), stabilization of the utrophin-glycoprotein complex (UGC) through biglycan or GALGT2 (**C**), or counteracting *UTRN* mRNA degradation by blocking microRNAs (miRNAs; (**D**) with AONs (1) or by the CRISPR/Cas9-directed excision of the DNA sequence, which upon transcription serves as a binding site for miRNAs (2)

Nonsense mutations can also be corrected through the CRISPR/nCas9 base editing method. For example, Ryu et al. showed that with the base editor-induced substitution of adenine to guanine, it is possible to exchange a premature stop codon for glutamine and obtain the full-length dystrophin translation in 17% of myofibers of the injected TA of dystrophic mice [168]. In a more recent study, Xu et al. showed that modified variants of adenine base editor (iABE–NGA) improved the editing efficiency and specificity [167]. The optimalization of the PAM-interacting domain allowed for nearly complete rescue of dystrophin in *mdx*4cv mouse hearts, with up to 15% rescue in skeletal muscle fibers. Furthermore, low off-target effect and no toxicity were detected. The functionality of the CRISPR/Cas9 prime editing method was also confirmed in iPSC-derived cardiomyocytes carrying an exon 51 deletion in the *DMD* gene [172]. This approach enabled insertion of two base pairs in exon 52, which led to restoration of the ORF and translation of the truncated dystrophin at 24.8–39.7% of the control level. Additionally, the corrected cardiomyocytes showed improvement in contractile functions.

Some studies, however, point to limitations of the CRISPR/Cas9-mediated editing of the *DMD* gene. Particularly, potentially therapeutic excision of exons 6 and 7 in a mouse model carrying an out-of-frame deletion mutation of exons 8–34 resulted in synthesis of corrected mRNA but only low-level production of truncated dystrophin, presumably due to low protein stability [173]. In contrast, an increased amount of Dp71 variant with an altered C-terminus (Dp71f variant, without exon 78) was observed at the sarcolemma, shown previously to modify the dystrophin function [174]. Furthermore, nonuniform correction of the *DMD* gene after the CRISPR/Cas9-mediated exon excision across nuclei in myofibers resulted in poor therapeutic efficacy, despite the overall high dystrophin protein levels, likely because of segmental reparation of the sarcolemma [175, 176]. Morin et al. indicated that modest but uniform distribution of dystrophin at the membrane of myofibers, obtained e.g., with AONs, might be more therapeutic than high levels of dystrophin generated only in some nuclei in the myofiber [175].

Although genome editing therapies are theoretically capable of producing the intended therapeutic effect after a single application, retreatment may be necessary for patients with DMD [177]. Losing Cas9 and gRNA after the desired change has occurred in myofibers would not be problematic as the target site would be corrected. However, since these changes most often result in production of only a partially functional protein, this will not fully prevent the muscle damage associated with DMD. Moreover, when severe damage occurs, the multinuclear muscle fiber can lose a nucleus containing the therapeutically altered genetic material, which might lead to restoration of the original mutation. Therefore, patients receiving this form of therapy might experience a progressive decline in dystrophin levels unless the CRISPR/Cas9 is delivered efficiently to satellite cells.

Another aspect that adversely affects CRISPR/Cas9-based therapies is the possibility of genome editing in nondesired sites (off-target mutations). This can be particularly worrying in the case of in vivo editing as it can lead to gene dysfunction, epigenetic changes, or even carcinogenesis [177]. However, many studies indicate a low risk of this process and given that the muscle tissue can be considered permanently post-mitotic, off-target mutation oncogenic effects are unlikely. Moreover, there are methods to reduce chances of such side-effects without sacrificing efficient genome editing, for example, by using a pair of nCas9 to generate two incisions instead of DSBs or by using the FokI–dCas9 system [178].

As in the case of therapies involving in vivo delivery of micro- or mini-dystrophin genes, there are also risks associated with CRISPR/Cas9 delivery by rAAV vectors. The possible immune response of the organism against rAAV, a therapeutic protein, as well as Cas9, could prevent the desired outcome [179, 180]. An antinuclease immune response may be induced in part by prior exposure of the organism to the bacteria from which Cas9 is derived. Because of this response, cells of the immune system could eliminate Cas9-containing cells, rendering the therapy futile. One of the proposed solutions is to use a vector suppressing the body’s immune response [124] which allowed for effective expression of the mini-dystrophin gene in double-knockout *mdx*/mTR^G2^ mouse model. Also, special immunosuppression and specially designed dystrophins are in tests [130, 181].

## Utrophin-Based Therapies

Studies originally performed in a mouse model of DMD demonstrated enormous potential of both the full-length and truncated utrophin in compensating for the lack of dystrophin and preventing muscular dystrophy [68, 182]. Furthermore, increased full-length utrophin synthesis ameliorated the pathology in a dose-dependent manner [68], without any toxicity [183], and truncated utrophins mitigated the pathology in dystrophic mice and dogs [69, 184]. Surprisingly, recent studies indicate that while dystrophin and utrophin can co-exist at the sarcolemma [185] and μDys is stably localized at distinct types of myofibers for a long period of time in *mdx* mice, μUtr is gradually waning from dystrophin-deficient 1, 2a, and 2d types of fibers, in which higher expression of the *utrn* gene is observed [186]. Further research is required to address this discrepancy and relevance to human clinical studies. It is important to note, however, that like dystrophin, also truncated utrophin sequences might require optimization to, e.g., reduce its stiffness, alter the mode of interaction with actin filaments, increase the affinity to β-dystroglycan, or add fragments necessary for interaction with crucial proteins, such as nNOS or MARK2 [20, 39, 45, 74, 76].

Apart from approaches based on delivery of micro-utrophin genes [107, 112, 186, 187] (Fig. 7A), strategies grounded on overexpression of the native *UTRN* gene through small molecules or peptides, including orally bioavailable ezutromid (formally SMT C1100) and SMT022357 [66, 188], heregulin [189], or activators of the NO pathway [190], as well as the use of genetic engineering tools such as artificial zinc finger transcription factors “Jazz” and the updated version “JZifi1” [191, 192] or CRISPR/Cas9 [193] can be distinguished. Furthermore, there are strategies used to increase the utrophin protein level at the sarcolemma and stabilize the UGC [194–196] or prevent degradation of the *UTRN* transcripts [197–199].

One of the prospective strategies to boost the utrophin levels is activation of *UTRN*-A promoter with orally available drug ezutromid or SMT022357, the second-generation compound with improved physicochemical properties [188] (Fig. 7B). Daily doses of ezutromid increased the expression of the *utrn* gene in striated muscles of *mdx* mice, resulting in sarcolemmal stability and amelioration of the dystrophin loss-associated pathology [200]. Ezutromid went successfully through phase 1 (healthy male volunteers) and phase 1b (DMD boys) clinical trials that tested its safety [201, 202]. Nonetheless, a phase 2 trial failed to achieve its endpoints and the development program has been abandoned by Summit Therapeutics [203].

Changes in gene activation can be induced by administration of complexes composed of catalytically inactive dCas9 with transcription activator or repressor [204, 205]. To increase the expression of the *UTRN* gene, dCas9 might be bound to multiple copies of VP16 transcriptional activator, and such complex could be then directed to *UTRN-A* and *-B* promoters by specially designed gRNAs, where VP16 domains would facilitate recruitment of the pre-initiation complex. Such approach led to elevated expression of the *UTRN* gene in myoblasts derived from a DMD patient with exon 42–52 deletion [206]. In a more recent approach that overcomes the large size of dCas9 fused to a transcriptional activation domain, a dual-rAAV9 system was successfully used to elevate the utrophin levels and ameliorate muscular dystrophy in *mdx* mice [193]. Although this is still an experimental strategy that requires further research, it could be of great benefit in treating both DMD and BMD patients, leading to a sustained increase in the expression of the utrophin gene, regardless of the type of the mutation in the *DMD* gene [207].

Another molecule shown to increase the utrophin level in membrane fractions in muscle cells is biglycan (Fig. 7C). Biglycan is a leucine-rich protein of the extracellular matrix (EMC). Following synthesis, it plays an important role in muscle development and regeneration, localizing dystrobrevins, syntrophins, and nNOS to the sarcolemma and stabilizing the DGC [208, 209]. Biglycan comes in two forms with significantly different functions. While the glycanated form does not seem to have a therapeutic value in DMD, the intraperitoneal injection of recombinant human nonglycanated biglycan upregulated utrophin, sarcoglycans, and nNOS, as well as improved the overall muscle health and function in *mdx* mice [196]. The nNOS increase at the sarcolemma of dystrophic myofibers might be unexpected given the results obtained from other studies [45] and the fact that utrophin does not contain the site present in dystrophin (R16–R17) that interacts with syntrophin/nNOS [46, 72] and might indicate that nNOS might be localized to the sarcolemma through mechanisms independent of utrophin/dystrophin. Biglycan was also upregulated in *mdx* mice by the rAAV vectors. Particularly, intravenous delivery of rAAV8 carrying biglycan cDNA resulted in increased utrophin levels as well as γ-sarcoglycan, α-dystrobrevin, and α1-syntrophin [210]. An optimized nonglycanated version of biglycan for systemic delivery to humans developed by Tivorsan Pharmaceuticals, called TVN-102, is currently in the preclinical testing phase [211].

Cytoxic T cell GalNAc transferase (Galgt2) is an enzyme normally distributed at NMJs of myofibers that can glycosylate α-dystroglycan in extrasynaptic regions when upregulated in wt and *mdx* myofibers [194, 212]. *Mdx/Galg2* transgenic mice displayed no signs of muscular dystrophy attributed to increased utrophin and other UGC components at the sarcolemma [194] (Fig. 7C). Interestingly, reduced myofiber diameter presumably due to inability of satellite cells to fuse with myofibers and abnormalities in NMJs were observed upon embryonic upregulation of Galgt2 [194, 212]. These disadvantageous effects can be omitted in mice by postnatal delivery of *GALGT2* with rAAV; however, these entitle some therapeutic effects that are utrophin independent [195, 213]. rAAVrh74/*GALGT2* delivery to gastrocnemius in macaques also resulted in increased glycosylation of α-dystroglycan and elevated levels of utrophin [214], which initiated a phase 1/2 clinical trial assessing the safety and efficacy of *GALGT2* gene therapy in humans (NCT03333590). The most recent data from dystrophic dogs that were administered intravenously with rAAVrh74/*GALGT* and analyzed 3 months later indicate that while the treatment induces muscle glycosylation, *UTRN* expression and lowers fibrosis, it has no effect on muscle strength [215]. Based on this data, the authors indicate that the *GALGT2* therapy might be better suited for younger individuals before the onset of the severe form of the disease.

An alternative solution to enhance the expression of the *UTRN* gene would be to block the repression of mRNA transcripts (Fig. 7D). As previously mentioned, utrophin occurs naturally in myofibers; however, during muscle differentiation, its expression is attenuated and dystrophin replaces utrophin at the sarcolemma [216]. Apart from the direct negative regulation of the promoter [217], posttranscriptional repression mechanisms targeting the utrophin mRNA significantly reduce the expression of the *UTRN* gene [218]; hence, blocking this process would have a significant effect on the amount of utrophin protein. Indeed, Loro et al. identified trichostatin A (TSA) and giovinostat as two compounds that could relive utrophin posttranscriptional repression based on a high-throughput screen with 3127 particles, 1000 of which were FDA-approved drugs, and a reporter containing *UTRN* 5′ and 3′ UTRs [197]. Interestingly, their potential to support the treatment of DMD has been previously discovered; however, their mechanism of action was linked to their ability to inhibit histone deacetylases [219, 220]. Other strategies aiming at posttranscriptional control of the expression of the *UTRN* gene include blocking miRNAs with AONs [198] (Fig. 7D1) and cutting out the 3′ UTR region targeted by miRNAs in the *UTRN* gene by CRISPR/Cas9 [199] (Fig. 7D2).

The use of utrophin in the treatment of DMD and BMD is a promising solution. It is important to note, however, that the regulatory machinery of the *UTRN* gene expression appears to be very complex. Recent results by Georgieva et al. point out that the epigenetic manipulation of the downstream utrophin enhancer is yet another promising approach to increase the utrophin levels and improve the muscle function [221]. Certainly, utrophin cannot completely compensate for the lack of dystrophin, which is especially apparent when dystrophic muscles with high amounts of utrophin are forced to exercise [45]. This might be due to its different mechanistic properties [16, 17] and/or the lack of ability to bind specific proteins such as nNOS [45]. Nevertheless, neither mini- nor micro-dystrophins can compensate for the lack of the full-length dystrophin as observed in BMD patients. An important aspect that may outweigh the advantages associated with therapies based on dystrophin gene repair or delivery, is that utrophin does not induce an immune response in DMD patients. Furthermore, utrophin-based therapies can be used regardless of the mutation type in the *DMD* gene.

## Conclusions

Corticosteroids, mechanical ventilation, cardiac medication, and rehabilitation markedly raised the median life expectancy of DMD patients born in recent years [130, 222]. In the meantime, many experimental therapeutic approaches have been advanced, with some of them shown to inhibit the disease progression not only in distinct disease animal models but also in humans. First new drugs based on restoration of the reading frame of dystrophin via readthrough of nonsense codons, antisense oligonucleotide-driven exon skipping, and rAAV-mediated delivery of μDys are now available to patients in some countries. The first two strategies are limited to DMD boys with strictly defined mutations and induce dystrophin translation in a relatively small number of fibers. However, even this limited therapeutic outcome has the potential to ameliorate the disease progression and prolong their lives. On the other hand, the recently approved treatment based on rAAV–μDys–ST can be theoretically applied to all patients regardless of the *DMD* genetic alteration and has the potential to induce a high level of the therapeutic protein in a vast number of fibers. Refinement of the above strategies as well as others, grounded on, e.g., drugs designed to activate transcription of the *UTRN* gene or stabilize the UGC is ongoing [223].

The structural and functional properties of both dystrophin and utrophin proteins have a crucial impact on the therapeutic outcome. Theoretically, delivery of therapeutics based on repair or restoration of the *DMD* gene expression should be more beneficial to patients than strategies based on μUtr synthesis or upregulation of the *UTRN* gene. However, dystrophin is not naturally available in DMD boys and immune response against newly synthesized dystrophin might render such therapeutic approaches futile [123]. Careful comparative analyses of dystrophin and utrophin might lead to the design of safer and more therapeutic dystrophins, with proper folding characteristics. Particularly, we show that H1 and H3 regions that have been commonly used in μDys share no sequence homology to the corresponding regions in utrophin that is naturally present in dystrophic tissues, and thus could, theoretically, more easily induce the immune response in treated DMD boys. On the other hand, despite the sequential and structural limitations, utrophin can partially compensate for the lack of dystrophin in animal models of DMD. It is important to stress though that long-term outcomes of therapeutic strategies grounded on utrophin and engineered micro-proteins are currently unknown and require further clinical testing.

Every presented therapeutic approach, while beneficial, has its own limitations. It is known that dystrophin and utrophin can co-localize at the sarcolemma [185]. The moderate level of utrophin does not affect the *DMD* gene expression, but its high amounts lead to a reduction of dystrophin, suggesting that there are finite β-dystroglycan and actin binding sites at the muscle membrane and in the interior side of muscle cells, respectively. Nevertheless, the ability of utrophin and dystrophin to co-localize sheds light on the possibility of combined dystrophin/utrophin therapy. Guiraud et al. showed that 30% AON restoration of dystrophin and overexpression of utrophin can lead to greater therapeutic benefits than every single approach alone [185]. The data suggest that combining various therapies might be a better solution, overcoming limitations of each approach.
